# Laser-plasma-based Space Radiation Reproduction in the Laboratory

**DOI:** 10.1038/srep42354

**Published:** 2017-02-08

**Authors:** B. Hidding, O. Karger, T. Königstein, G. Pretzler, G. G. Manahan, P. McKenna, R. Gray, R. Wilson, S. M. Wiggins, G. H. Welsh, A. Beaton, P. Delinikolas, D. A. Jaroszynski, J. B. Rosenzweig, A. Karmakar, V. Ferlet-Cavrois, A. Costantino, M. Muschitiello, E. Daly

**Affiliations:** 1 SUPA, Department of Physics, University of Strathclyde, Glasgow, UK; 2 Institut für Experimentalphysik, University of Hamburg, Germany; 3 Institute for Laser and Plasma Physics, Heinrich-Heine-University Düsseldorf, Germany; 4 University of California, Los Angeles, USA; 5 Leibniz Supercomputing Centre, Boltzmannstr. 1, 85748 Garching, Germany; 6 European Space Agency, Noordwijk, Netherlands

## Abstract

Space radiation is a great danger to electronics and astronauts onboard space vessels. The spectral flux of space electrons, protons and ions for example in the radiation belts is inherently broadband, but this is a feature hard to mimic with conventional radiation sources. Using laser-plasma-accelerators, we reproduced relativistic, broadband radiation belt flux in the laboratory, and used this man-made space radiation to test the radiation hardness of space electronics. Such close mimicking of space radiation in the lab builds on the inherent ability of laser-plasma-accelerators to directly produce broadband Maxwellian-type particle flux, akin to conditions in space. In combination with the established sources, utilisation of the growing number of ever more potent laser-plasma-accelerator facilities worldwide as complementary space radiation sources can help alleviate the shortage of available beamtime and may allow for development of advanced test procedures, paving the way towards higher reliability of space missions.

Radiation hardness assessment of onboard electronics – an essential part of every space mission – is ideally achieved by reproducing the mission-specific space radiation environment as accurately as possible^1^,^2^. Testing in space would be the most realistic method, but is mostly cost prohibitive. Conventional accelerators such as linacs and cyclotrons are used instead, which produce well defined, but monoenergetic electron, proton and ion flux. However, space radiation for example in the form of “killer” electrons^3^,^4^,^5^ is very broadband, generally describable by power law or exponential functions^2^. While the production of monoenergetic beams is challenging with laser-plasma-accelerators^6^,^7^,^8^,^9^,^10^ (LPAs), in contrast producing broadband radiation is the inherent regime of LPAs^11^,^12^, a unique ability which could be exploited for the benefit of the space radiation testing community^13^,^14^,^15^.

## Results

**Figure 1:**
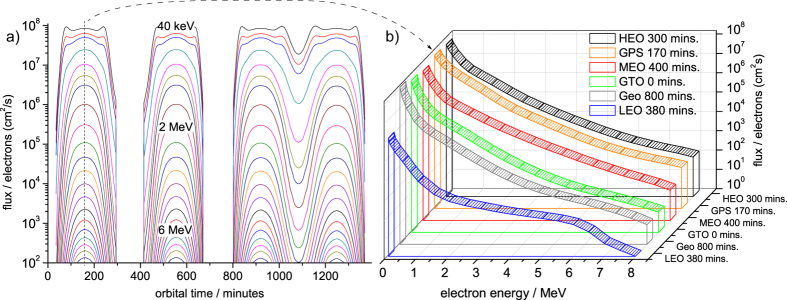
Electron flux in the inner van Allen belt according to the NASA AE9 model at various orbits. In (**a**), the flux on GPS orbit is given via contour plots as a function of orbital time, and in (**b**) the maximum spectral flux on various orbits from LEO to HEO is plotted, demonstrating the broadband energy range up to *E* ~ 10 MeV. The GPS electron spectrum at maximum flux from (**a**) is shown in (**b**) with the orange plot, and is used as a showcase for the experiments. The flux axis is logarithmic, indicating that the shape can often be approximated by exponential distribution functions.

Here we report on experiments, in which broadband space-level electron and proton flux was produced with LPAs having peak laser powers in the *P* ~ 150 TW to PW range. NASA’s AE8/AP8 and AE9/AP9 models^16^ were used to calculate the typical electron and proton flux at different orbits in the van-Allen belts. As a showcase, the electron spectral flux at the important GPS satellite orbit in the outer van-Allen belt is given in Fig. 1(a).

**Figure 2:**
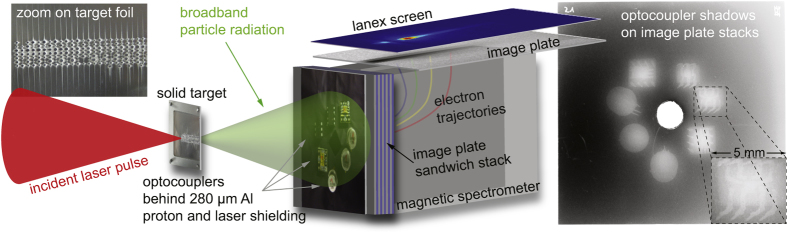
Experimental setup with 150 TW Ti:sapphire laser. The laser-solid-interaction produces broadband, broad-angle particle radiation with electrons in the 1–10 MeV range; protons are eliminated by a thin protection foil directly in front of the DUTs (not shown here, see methods). The electrons irradiate optocouplers (located 5 cm away from the target foil) and are then detected on an image plate stack to retrieve the spatially resolved temperatures and divergence. The high-resolution shadow of the optocouplers on a front image plate is demonstrated on the right hand side; single optocoupler pins and detailed internal structure of the devices are clearly resolved. A central hole allowed on-axis electrons to enter a permanent magnet spectrometer where simultaneous measurement was obtained on an additional image plate, and a Lanex scintillating screen.

To reproduce this broadband van-Allen belt level electron spectral flux, a university lab scale Ti:Sapphire laser^17^ at a power level of *P* ~ 150 TW was used. The laser pulses were focused to spot sizes in the range of a few μm^2^ on thin metal foil targets, corresponding to interaction intensities of *I* ≈ 10^18^–10^20^ W cm^−2^. Such laser-overdense plasma interaction is one of the most effective and reliable methods to convert laser energy into broadband electron flux, and also into protons via the TNSA mechanism^18^. In this scenario, it is well known that the resulting energy *E* of the accelerated electrons can be approximated by an exponential distribution *N* = *N*_0_ exp(−*E*/*k*_B_*T*), where *N* is the number of electrons, *k*_B_ is the Boltzmann constant and *T* the electron temperature. Established scalings by Wilks^19^, Beg^20^ and Kluge^21^, refined by particle-in-cell-simulations, were used to predict the effective temperature *T*_eff_ = *k*_B_*T* as a function of laser intensity on target. By adjusting the laser intensity to values of *I* ≈ few 10^19^ W cm^−2^, the exponential electron flux was tuned to match the electron spectrum as present in the van Allen belt, e.g. *T*_eff_ ≈ 0.6 MeV on the GPS orbit. Figure 2 illustrates the experimental setup. The laser–foil interaction produces broadband particle radiation, which was monitored with state-of-the-art diagnostics and was used to irradiate various commercial and radiation-hardened optocouplers as devices under test (DUT), see Methods section.

**Figure 3:**
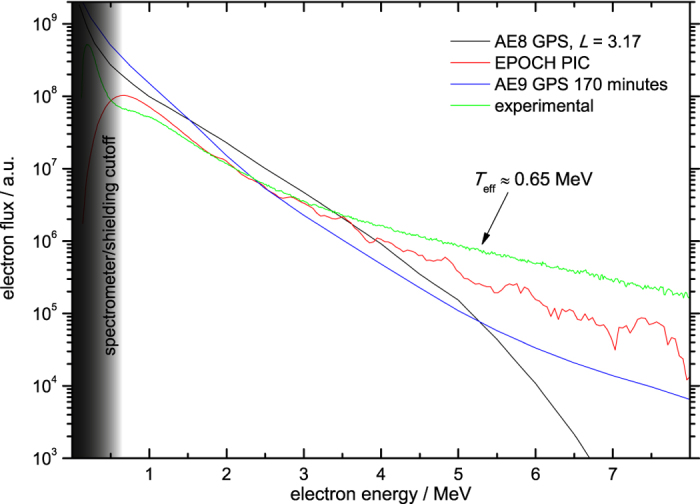
Calculated (black (AE8) and blue (AE9) lines), PIC-simulated (red) and experimentally obtained (green) electron flux for GPS orbit. By fine-tuning the laser-plasma-interaction at an intensity *I* ≈ 4.5 × 10^19^ W cm^−2^ at an incidence angle of 45°, the experimentally obtained electron spectral flux was tuned to match the space-borne van-Allen belt spectral flux.

Figure 3 demonstrates the successful reproduction of GPS-level electron flux at a laser intensity *I* ≈ 4.5 × 10^19^ W cm^−2^, producing electron flux with *T*_eff_ ≈ 0.65 MeV. The agreement is especially good at the medium energy range, which is particularly important, whereas the number of high energy electrons is low due to the exponential decrease, and the numerous low energy electrons on the other hand would be absorbed by the spacecraft shielding.

**Figure 4:**
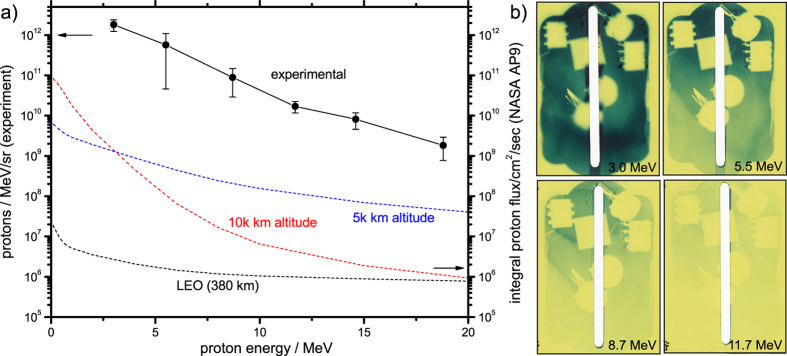
Results of proton irradiation at VULCAN. (**a**) compares the retrieved proton flux (solid black line, left y-axis) with the broadband flux at LEO and at higher altitudes of 5 k and 10 k km predicted by the AP9 model (dashed lines, right y-axis), and (**b**) shows the raw radiography images generated by the proton flux on the proton-sensitive radiochromic film (RCF) stack.

A second set of campaigns at the VULCAN PW-laser aimed at the production of broadband space protons. At these much higher laser pulse powers, protons with energies up to *E* ≈ 20 MeV were generated and used to irradiate a further set of optocouplers, as shown in Fig. 4. Again, the measured spectra retrieved from radiochromic film stacks have exponential slope and are particularly useful to reproduce certain similar space spectral flux on various orbits ranging from LEO to the proton-rich inner van-Allen belt at 5 k km altitude and beyond.

**Figure 5:**
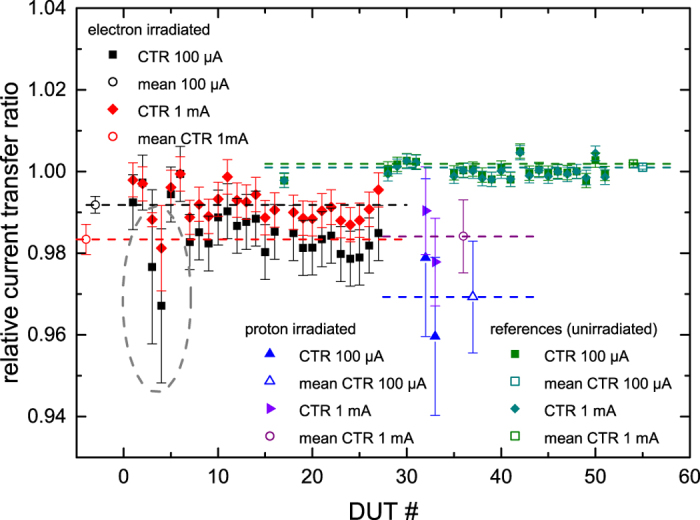
Optocoupler performance degradation after laser-plasma-produced electron and proton irradiation. Shown is the current transfer ratio (CTR) degradation of Vishay SFH6345 optocouplers after irradiation with van-Allen belt type electron fluence of 3 × 10^9^ e-/cm^2^ up to 7 × 10^9^ e-/cm^2^ (dashed grey ellipse) and proton fluence of 5.3 × 10^10^ p-/cm^2^, demonstrating significant degradation when compared to unirradiated benchmark optocouplers.

The irradiation of the optocouplers by reproduction of either the outer van-Allen belt electron flux or the inner van Allen belt proton flux has led to significant degradation of optocoupler performance, making use of state-of-the-art testing procedures adapted from the European Space Agency. This is shown in Fig. 5, where the current transfer ratio (CTR) of Vishay SFH6345 optocouplers after applying broadband electron and proton fluences of up to ≈7 × 10^9^ e-/cm^2^, and ≈5.3 × 10^10^ p-/cm^2^, respectively, shows degradation of up to 4% when compared to unirradiated reference optocouplers.

## Discussion

We have shown that LPAs can be used to accurately reproduce broadband inner- and outer van Allen belt electron and proton flux, and we used this lab-made space radiation to systematically characterise realistic degradation of space electronics. These measurements demonstrate for the first time that laser-plasma-accelerators are viable tools for space radiation testing and add a novel capability to the arsenal of ground-based testing techniques. It is expected that the steady rapid progress in laser-plasma accelerator technology will allow the energy and flux range of accurately reproducible space radiation to be further extended. For example, the radiation belts of other magnetised planets such as Jupiter, Saturn, Uranus and Neptune^22^ are also populated with energetic electrons, protons and ions. Because the magnetic field of these planets is partially much stronger than that of the Earth, much higher energy electrons are generated, in case of Jupiter, as far as is known typically up to 50 MeV. Exploratory missions in this harsh Jovian radiation environment have a high scientific priority, for example because of the possibility of water on Io^23^. While such energy distributions can also be reached with laser-overdense interaction using higher laser energies and intensities, this higher electron energy range is better accessible when using underdense, gaseous targets^24^. Improvements both on the target side (e.g. tape drives for quasi-continuous irradiation) and laser technology side (e.g., kHz repetition rate systems) will furthermore lead to increased accelerator repetition rates and therefore to higher average flux and in turn to accelerated testing times. Higher repetition rates also allow to decrease peak flux levels, or in the other extreme high peak fluxes (close to target) could be useful to study collective or nonlinear radiation effects^15^. As chip structure size continues to decrease, single event effects (SEE) in microelectronics generated by electrons are gaining importance^25^,^26^. In parallel, also lower energy protons and ions are attaining increased interest for space radiation effects^27^. Both electrons in a wide energy range up to hundreds of MeV, and lower to moderate energy protons (few to tens of MeV-scale) and ions are very well accessible by today’s typical LPAs of the few hundred TW class. Record values for peak electron energies obtainable with LPA’s exceed ~4 GeV^28^, and can reach the hundred MeV level for protons with today’s highest power laser systems. While for example the South Atlantic Anomaly (SAA) generates low-altitude protons of energies exceeding this current limit considerably^29^, such proton energy levels are increasingly accessible with LPA’s, too^30^, although at the cost of repetition rate. Finally, while the reported experiments have been carried through with electronics as DUTs, space radiobiology, a topic which has recently been highlighted during Orion spacecraft’s Exploration Test Flight-1, can also be studied with LPAs, and we believe that the accurate reproduction of space radiation will contribute substantially to increase future mission reliability and safety.

## Methods

### Electron production, measurement and irradiation at Arcturus 150 TW-laser

Ti:sapphire laser pulses with the Arcturus laser system at University of Düsseldorf were used with pulse durations down to 23 fs and effective pulse powers up to ~150 TW to irradiate thin ~25 μm Al foils after being strongly focused to spot sizes down to ~6 μm^2^ with an f/2 parabola, yielding intensities of up to *I* ≈ 8 × 10^19^ W cm^−2^. The energy deposition on the target Al foil of thickness ~25 μm eventually leads to local melting of the foil material (see Fig. 2, top left), and the target foil is therefore shifted to a new fresh spot after each shot with a translation stage to provide an uncompromised surface. All off-axis protons are filtered out by a ~280 μm thick aluminum foil (not shown in the figure) directly in front of the DUT’s, which are located 5 cm downstream of the interaction point. The electron beam output was tuned by changing the laser energy, the intensity on target by moving the target relative to the focus (Rayleigh length z_R_ ~ 30 μm), and the incidence angle (mostly at 45°). The laser-plasma interaction scalings predict that a laser intensity on target in the range of *I* ≈ few 10^19^ W cm^−2^ is suitable to reproduce the exponential space electron flux with temperatures *T*_eff_ ≈ 0.6 MeV. To refine these predictions, particle-in-cell simulations were carried out. The on-axis electron spectrum was measured by a state-of-the-art permanent magnet based spectrometer using image plates and Lanex screens, which were monitored with CCD cameras as online diagnostics. An 8 cm × 8 cm area image plate stack similar as in ref. 30 in target normal direction behind the optocouplers provided detailed information on the divergence of the produced electron beams and the integrated fluence. The divergence amounted to approximately 600 mrad for lower energy electrons down to nearly 400 mrad for higher energy electrons. Various other diagnostics (further electron spectrometers and image plate stacks) were used to monitor electron flux in addition to the target normal direction to give a nearly 360° radiation topology (not shown). To increase the efficiency of the radiation source, these other directions which partially exhibit substantial flux values of broadband radiation, can also be harvested for DUT irradiation.

### Proton production, measurement and irradiation at VULCAN PW

The VULCAN laser at Rutherford Appleton Laboratory operated at an energy of ~150 J on target, delivering a peak intensity on target of *I* ≈ 4 × 10^20^ W/cm^2^ at an incidence angle of 0°. A plasma-mirror was used to reduce the preplasma on the target foil, which was 20 μm thick Cu. Measurement of the proton flux was performed using stacked radiochromic dosimetry film (RCF), consisting of (front to back) 13 μm Al protective filter foil, 63 μm Al optocoupler mount and filter foil, followed by RCF film and Mylar filters suitable to resolve the proton spectrum (see Fig. 4).

### Particle-in-cell simulations

2D PIC-simulations where carried through with the code EPOCH in the PICS Laboratory Astro at Leibniz Computing Centre Munich, with a simulation box size of 40 μm × 20 μm and a 4000 × 1000 grid. The simulated interaction represents the experimental setup with a laser pulse duration of *τ* = 23 fs and a central wavelength of λ = 0.8 μm being focused to a spot size of *w*_0_ = 3 μm on 30 μm thick Al at an incidence angle of 45°. The target was modeled by a tiny exponential ramp of 0.5 μm thickness to account for the preplasma, then ramping up to 30 times critical density n_c_. A range of intensities have been scanned in various runs, and the electron spectrum depicted in Fig. 3 which fits best the GPS spectrum is obtained at an intensity of *I* = 4.5 × 10^19^ W cm^−2^.

### Optocoupler handling and performance testing

Various types of optocouplers were used as DUTs in the experiments. Shown here is the performance degradation of Vishay SFH6345 8-pin optocouplers, using the current transfer ratio (CTR) between input and output current. The devices partially received different amounts of shots. Testing device #3 and #4 were irradiated with 478 electron pulses in total, the highest amount of shots of all tested devices, thus showing the most significant decrease in the current transfer ratio. The error bars in the figure represent the standard deviation from the mean value. This key performance parameter was measured before and after irradiation campaigns in a climatised environment at ESTEC, using an Agilent Test Fixture 16442A in combination with the precision semiconductor parameter analyzer Agilent 4156C. The CTR was measured at input currents of 100 μA and 1 mA, respectively. Non-irradiated optocouplers were used as reference. The performance testing was conducted in accordance with state-of-the-art ESA testing standards.

### Data Availability

The output data from this research is available and can be accessed at: http://dx.doi.org/10.15129/be393aa3-9e35-47c5-aae4-b2444dfb7893.

## Additional Information

**How to cite this article**: Hidding, B. *et al*. Laser-plasma-based Space Radiation Reproduction in the Laboratory. *Sci. Rep.* **7**, 42354; doi: 10.1038/srep42354 (2017).

**Publisher's note:** Springer Nature remains neutral with regard to jurisdictional claims in published maps and institutional affiliations.
